# Bridging Randomized Trial Efficacy and Real‐World Effectiveness in Multiple Myeloma: Integrating Clinical Trials and Real‐World Evidence for Individualized Care

**DOI:** 10.1111/ejh.70263

**Published:** 2026-07-09

**Authors:** Enrica Antonia Martino, Ernesto Vigna, Antonella Bruzzese, Nicola Amodio, Santino Caserta, Eugenio Lucia, Graziella D'Arrigo, Virginia Olivito, Caterina Labanca, Francesco Mendicino, Maria Eugenia Alvaro, Giovanni Tripepi, Fortunato Morabito, Massimo Gentile

**Affiliations:** ^1^ Department of Onco‐Hematology AO of Cosenza Cosenza Italy; ^2^ Department of Experimental and Clinical Medicine University of Catanzaro Catanzaro Italy; ^3^ Istituto di Fisiologia Clinica del CNR di Reggio Calabria, Consiglio Nazionale Delle Ricerche Reggio Calabria Italy; ^4^ Dane Srl Reggio Calabria Italy; ^5^ Department of Pharmacy, Health and Nutritional Science University of Calabria Rende Italy

## Abstract

Multiple myeloma (MM) is predominantly a disease of older adults, yet the randomized clinical trials (RCTs) that define standards of care are conducted largely in younger, fitter, and less comorbid populations. This creates a systematic mismatch between the populations generating evidence and those receiving treatment in routine practice, which can be characterized by comparing eligibility criteria, baseline characteristics, treatment exposure, and outcomes across RCTs and large real‐world cohorts. Real‐world data (RWD) have emerged as an essential complement to RCTs, capturing treatment effectiveness, tolerability, and patterns of care in unselected populations. Still, their use in clinical decision‐making remains inconsistent. In this review, we use MM as a model to examine the divergence between trial efficacy and real‐world effectiveness. Outcomes observed in RCTs are reproducible primarily in patients who resemble trial populations. In contrast, in older, frail, and comorbid patients, effectiveness is frequently attenuated by increased toxicity, reduced dose intensity, and early treatment discontinuation. We summarize how differences in comorbidity burden, frailty status, treatment intensity, and early discontinuation contribute to attenuated outcomes in routine care. Frailty, rather than chronological age alone, appears to be the principal determinant of this divergence. Despite its strong prognostic and predictive value, frailty is inconsistently measured in both RCTs and RWD, limiting the translation of evidence into practice. We highlight the prognostic and predictive value of formal frailty assessment and its current under‐use in both RCTs and RWD. On this basis, we propose a pragmatic, patient‐centered approach that uses trial‐derived estimates of regimen efficacy together with real‐world data on toxicity, dose intensity, and treatment persistence to enable systematic adaptation of regimen choice, dose, and schedule. Finally, we outline methodological priorities for future research, including standardized data elements, robust causal‐inference approaches, routine incorporation of frailty, and closer alignment between RCTs and RWD. Although focused on MM, this framework has broader implications across hematologic malignancies, where bridging the gap between efficacy and effectiveness is essential to ensure that therapeutic advances translate into meaningful benefit for patients seen in everyday clinical practice.

## 1 Introduction

Multiple myeloma (MM) is a prototypical hematologic malignancy in which the distance between “trial patients” and “real patients” is especially evident. The median age at diagnosis is around 70 years, and a substantial proportion of patients are over 75–80 years, frequently with multiple comorbidities, organ dysfunction, and varying degrees of frailty [1, 2, 3]. Yet the pivotal randomized controlled trials (RCTs) that establish new standards of care usually enroll younger, fitter individuals with strict organ function, performance status, and eligibility criteria, often treated in specialized centers with optimal supportive care [4, 5, 6, 7, 8, 9].

This creates a structural mismatch between the population generating evidence and the population receiving it. In the former, high‐quality RCTs define the efficacy and safety of increasingly complex regimens—triplets, quadruplets, monoclonal antibodies, cellular therapies—under controlled conditions [4, 5, 6, 7, 8, 9, 10, 11]. Importantly, RCTs primarily provide estimates of comparative efficacy, including the relative magnitude of benefit between therapeutic strategies, rather than directly predicting the absolute outcomes achievable in heterogeneous populations encountered in routine practice. In the latter, everyday practice confronts clinicians with older, frailer, multimorbid patients who resemble RCT participants only partially and who often cannot tolerate full‐intensity regimens for prolonged periods [1, 2, 3, 10, 11, 12].

Real‐world data (RWD) and real‐world evidence (RWE) have emerged as crucial complements to RCTs, offering insights into treatment effectiveness, tolerability, and patterns of care in unselected populations [13, 14, 15, 16, 17, 18, 19, 20, 21, 22, 23, 24, 25, 26, 27]. However, RWD are heterogeneous in quality and design, often lack standardized endpoints, and are subject to multiple forms of bias [14, 15, 16, 17, 18, 19, 20, 21, 22, 23, 24, 25, 26, 27]. In MM, the rapid expansion of therapeutic options has not always been matched by equally robust realworld evaluation, particularly in very elderly and frail patients [12, 13, 14]. The increasing relevance of RWE has also influenced regulatory science. Regulatory agencies, including the U.S. Food and Drug Administration (FDA) and the European Medicines Agency (EMA), increasingly recognize high‐quality RWE as a complementary source of evidence for post‐marketing surveillance, long‐term safety assessment, and evaluation of treatment effectiveness in populations underrepresented in clinical trials. However, the regulatory acceptance of RWE requires robust methodologies, standardized data collection, and appropriate approaches to minimize bias [13, 14].

Although the focus is disease‐specific, the issues addressed—representativeness, external validity, frailty, and methodological rigor in RWD generation—are broadly relevant across hematology [13, 14, 15, 16, 17, 18, 19, 20, 21, 22, 23, 24, 25, 26, 27, 28].

In this review, we use MM as a model to examine the mechanisms underlying the gap between trial efficacy and real‐world effectiveness. We analyze how patient characteristics, including age, comorbidity burden, and frailty, influence treatment selection, toxicity, dose intensity, and clinical outcomes across RCTs and real‐world cohorts. We further discuss methodological approaches to integrate randomized evidence and observational data into a pragmatic framework for individualized therapeutic decision‐making.

## 2 Who is Really Represented in Pivotal Multiple Myeloma Trials?

Modern MM therapy has been shaped by a series of pivotal phase II and III randomized clinical trials (RCTs) across newly diagnosed transplant‐eligible (NDTE), transplant‐ineligible (NDTI), and relapsed/refractory (RRMM) settings. These studies have established the efficacy of proteasome inhibitor– and immunomodulatory drug–based combinations, monoclonal antibodies, and, more recently, cellular and T‐cell‐redirecting therapies [4, 5, 6, 7, 8, 9, 29, 30, 31, 32]. However, these trials primarily define therapeutic efficacy in selected populations and only partially reflect the demographic, biological, and functional heterogeneity of patients treated in routine practice [1, 2, 3, 10, 11, 12]. The main eligibility criteria and baseline characteristics of representative pivotal trials across transplant‐eligible, transplant‐ineligible, and relapsed/refractory MM are summarized in Table 1, highlighting the highly selected nature of trial populations compared with patients encountered in routine clinical practice.

**TABLE 1:** Key eligibility characteristics of pivotal randomized clinical trials in multiple myeloma.

Trial/regimen	Setting	Median age (years)	Patients ≥ 75 years	ECOG 0–1 (%)	Main eligibility features	Representativeness of routine practice
IFM 2009 (RVd ± ASCT) [4]	NDTE	60	0	NR	Age ≤ 65 years; transplant eligible; adequate organ function	Low
DETERMINATION (RVd ± ASCT) [5]	NDTE	57	0	> 90	Transplant eligible; ECOG 0–2	Low
CASSIOPEIA (D‐VTd vs. VTd) [6]	NDTE	59	0	NR	Transplant eligible; adequate organ function	Low
GRIFFIN (D‐RVd vs. RVd) [7]	NDTE	59	0	> 94	Age ≤ 70 years; transplant eligible	Low
FORTE (KRd‐based strategies) [8]	NDTE	57	0	NR	Age ≤ 65 years; transplant eligible	Low
FIRST (Rd vs. MPT) [9]	NDTI	73	35	~78	Transplant ineligible; ECOG 0–2	Moderate
SWOG S0777 (VRd vs. Rd) [29]	Mixed NDMM	62–65	NR	~86	Patients without immediate ASCT	Moderate
ALCYONE (D‐VMP vs. VMP) [30]	NDTI	71	30	~75	Transplant ineligible; adequate organ function	Moderate
MAIA (D‐Rd vs. Rd) [31]	NDTI	73	44	~83	Transplant ineligible; ECOG 0–2	Moderate
POLLUX (D‐Rd vs. Rd) [33]	RRMM	65	10	NR	1–3 prior lines; ECOG 0–2	Moderate‐Low
CASTOR (D‐Vd vs. Vd) [34]	RRMM	64	9	NR	1–3 prior lines; ECOG 0–2	Moderate‐Low
ASPIRE (KRd vs. Rd) [35]	RRMM	64	NR	> 90	1–3 prior lines; preserved cardiac function	Moderate‐Low
ENDEAVOR (Kd vs. Vd) [36]	RRMM	65	15	> 93	1–3 prior lines	Moderate‐Low
ELOQUENT‐2 (Elo‐Rd vs. Rd) [37]	RRMM	66	NR	> 90	1–3 prior lines	Moderate‐Low
TOURMALINE‐MM1 (Ixa‐Rd vs. Rd) [38]	RRMM	66	NR	> 93	1–3 prior lines	Moderate‐Low
KarMMa (ide‐cel) [38]	RRMM	61	NR	98	≥ 3 prior regimens; ECOG 0–1	Low
CARTITUDE‐1 (cilta‐cel) [39]	RRMM	61	NR	> 99	Heavily pretreated; ECOG 0–1	Low
MajesTEC‐1 (teclistamab) [40]	RRMM	64	≈15	> 95	Triple‐class exposed; ECOG 0–1; adequate organ function	Low
MonumenTAL‐1 (talquetamab) [41]	RRMM	61	58	93	Triple‐class exposed; ECOG 0–2; adequate organ function	Low

*Note:* Representative phase II and III trials across newly diagnosed transplant‐eligible (NDTE), transplant‐ineligible (NDTI), and relapsed/refractory multiple myeloma (RRMM). The table highlights the degree of patient selection with respect to age, performance status, and organ function, illustrating the limited representation of frail and multimorbid patients in pivotal randomized clinical trials.

Abbreviations: CrCl, creatinine clearance; ECOG, eastern cooperative oncology group performance status; HF, heart failure; HTN, hypertension; MI, myocardial infarction; NDTE, newly diagnosed transplant‐eligible; NDTI, newly diagnosed transplant‐ineligible; RRMM, relapsed/refractory multiple myeloma.

In NDTE trials, induction regimens and transplant‐based strategies—such as those evaluated in IFM 2009 and DFCI 10–106, as well as more recent quadruplet‐based approaches—are typically studied in relatively young and fit patients, with median ages in the late 50s to early 60s, excellent performance status (predominantly ECOG 0–1), and preserved organ function [4, 5, 6, 7, 8]. Individuals with significant cardiovascular disease, advanced renal impairment, or multiple uncontrolled comorbidities are generally excluded [4, 5, 6, 7, 8]. Thus, NDTE trials provide robust estimates of the benefit achievable with intensive approaches in selected patients but provide limited information on treatment feasibility among older patients with frailty, comorbidities, or reduced functional reserve.

Frontline trials in the transplant‐ineligible setting, including FIRST, SWOG S0777, ALCYONE, and MAIA, extend inclusion to older patients, with median ages in the early 70s [9, 29, 30, 31]. Nevertheless, these studies generally enrolled a biologically selected elderly population, with relatively preserved functional status and limited frailty. Consequently, even trials specifically designed for transplant‐ineligible patients largely represent fit older adults rather than the full spectrum of very elderly, frail, and multimorbid patients encountered in daily practice [9, 10, 11, 12, 29, 30, 32].

In the relapsed/refractory setting, pivotal studies of triplet combinations, monoclonal antibodies, and novel immunotherapies—such as POLLUX, CASTOR, ASPIRE, ENDEAVOR, ELOQUENT‐2, TOURMALINE‐MM1, and CAR‐T cell trials—impose additional selection constraints, including limits on prior lines of therapy, requirements for adequate hematologic and organ function, and exclusion of patients with uncontrolled comorbidities [33, 34, 35, 36, 37, 38, 39, 42]. While necessary to ensure safety and internal validity, these criteria limit the applicability of trial findings to heavily pretreated patients with cumulative toxicities, organ dysfunction, or impaired functional status, who represent a relevant proportion of real‐world RRMM populations [10, 11, 12].

When trial populations are compared with large registry‐based and observational cohorts, consistent discrepancies emerge. Real‐world patients are older, more frequently over 75–80 years, and carry a substantially higher burden of comorbidities, including renal impairment, cardiovascular disease, and other chronic conditions [1, 2, 3, 43, 44, 45, 46]. Performance status is more often impaired, and frailty—rarely captured in RCTs—is highly prevalent [47, 48, 49]. These differences translate into distinct therapeutic trajectories, including lower dose intensity, higher rates of toxicity, and earlier treatment discontinuation [10, 11, 12, 43, 44, 45, 46]. Importantly, attenuation of absolute outcomes in real‐world populations does not necessarily imply loss of relative treatment benefit. Comparative treatment effects observed in RCTs may remain consistent in broader populations, although the magnitude of absolute benefit is frequently influenced by patient characteristics, treatment intensity, and competing risks.

Taken together, these observations highlight a fundamental tension between internal and external validity. Pivotal RCTs in MM provide robust estimates of efficacy in selected populations under controlled conditions but they incompletely represent the patients most commonly encountered in clinical practice. Thus, pivotal trials in MM are internally valid and indispensable for defining treatment efficacy, but their external validity is limited by incomplete representation of the broader disease population.

## 3 Real‐World Multiple Myeloma Cohorts and Registries: What do They Capture?

Real‐world MM data arise from diverse sources, each with specific advantages and shortcomings. Population‐based cancer registries, national and international disease‐specific registries, cooperative‐group observational cohorts, transplant and cellular therapy registries, institutional databases, and administrative or claims datasets all contribute pieces to the overall picture [10, 11, 12, 13, 14, 15, 16, 17, 18, 19, 20, 21, 22, 23, 24, 25, 26, 27, 28, 43, 44, 45, 46].

Population‐based registries, such as national Scandinavian datasets, provide near‐complete capture of incident MM cases within defined geographic regions [44, 45]. Their principal strength lies in representing the full clinical spectrum of the disease, including very elderly patients and those with substantial comorbidity, across both academic and community settings. However, these registries often lack granular data on frailty, detailed treatment modifications, and standardized toxicity reporting.

Cooperative‐group and multicenter observational cohorts provide more detailed clinical information, including staging, cytogenetics, and treatment sequencing, and frequently include patients who would have been excluded from RCTs due to age, comorbidities, or prior therapies [31, 32, 46, 50, 51, 52]. These datasets are particularly informative for evaluating the real‐world effectiveness and tolerability of specific regimens. Nevertheless, they remain subject to center participation bias and may under‐represent the frailest or socially vulnerable patients.

Large international observational initiatives, including the CONNECT‐MM [53] and INSIGHT‐MM [54] studies, have provided important complementary information on treatment patterns, outcomes, sequencing strategies, and safety profiles across broad MM populations, including patients often excluded from clinical trials. Similarly, the PREAMBLE study has contributed prospective real‐world data on patient characteristics, treatment pathways, and outcomes in routine clinical practice [55, 56].

The principal sources of real‐world evidence in multiple myeloma and their complementary contributions to clinical research are summarized in Table 2.

**TABLE 2:** Major real‐world data sources and registries in multiple myeloma.

Dataset/registry	Region	Type of RWD source	Approximate population/period	Main contribution to MM real‐world evidence	Main limitations
Nordic Danish–Swedish national myeloma registries [44]	Denmark, Sweden	Population‐based national registries	> 10 000 patients; since ~2008	Near‐complete capture of incident MM cases; full age spectrum; long‐term survival; treatment patterns in unselected populations	Limited information on frailty, functional status, toxicity, and treatment modifications
Swedish population‐based cohorts/SEER‐Medicare datasets [3, 57]	Sweden/USA	Population‐based registry‐linked administrative datasets	Several thousand patients; contemporary treatment eras	Assessment of survival trends, healthcare utilization, treatment patterns, and outcomes in elderly populations	Limited clinical granularity; incomplete cytogenetics, response assessment, and frailty measures
CONNECT‐MM registry [53]	USA	Prospective multicenter observational registry	> 3000 patients; contemporary treatment era	Longitudinal characterization of treatment patterns, effectiveness, toxicity, and outcomes across the MM disease course, including patients under‐represented in trials	Participation bias; variable completeness of patient‐reported and clinical data
INSIGHT‐MM registry [54]	International	Large prospective global observational registry	> 4000 patients; contemporary treatment era	Real‐world treatment sequencing, effectiveness, safety, and outcomes across different healthcare systems; inclusion of broader patient populations	Heterogeneity of participating centers and treatment approaches
PREAMBLE study [55]	USA/international	Prospective observational cohort focused on real‐world treatment experience	Contemporary MM population	Detailed evaluation of patient characteristics, treatment pathways, outcomes, quality of life, and factors influencing treatment decisions	Limited follow‐up for some emerging therapies; observational design
UK Myeloma XI program [50, 51]	United Kingdom	Large multicenter clinical trial platform with pragmatic/RWE components	> 4000 patients; 2010s–present	Detailed clinical, genomic, treatment, and maintenance data; analyses of treatment strategies across risk groups	Not fully population‐based; frailest and very elderly patients remain under‐represented
European Myeloma Network (EMN) and cooperative‐group cohorts [18, 37, 38]	Europe	Multicenter observational cohorts	Hundreds–thousands; 2000s–present	Detailed clinical characteristics, cytogenetics, treatment sequencing, and outcomes with modern regimens	Center‐selection bias; heterogeneous data collection; incomplete frailty assessment
EBMT myeloma registry [58]	Europe/international	Transplant and cellular therapy registry	> 30 000 transplant procedures; 1990s–present	Real‐world outcomes after autologous transplantation; impact of age, disease risk, and transplant strategies	Includes only selected patients undergoing transplantation; limited information outside transplant pathway
CIBMTR myeloma registry [59]	International (mainly USA)	Transplant and cellular therapy registry	Tens of thousands; 1990s–present	Large‐scale evaluation of ASCT and CAR‐T outcomes, toxicity, and survival in routine practice	Selection of patients eligible for transplant/CAR‐T; limited capture of non‐cellular therapy populations
SEER‐Medicare and other claims‐based datasets [60]	USA	Administrative/claims databases	Large elderly populations	Healthcare utilization, treatment patterns, costs, survival, and outcomes at population scale	Limited disease‐specific variables; absence of standardized response, MRD, toxicity, and frailty assessments

*Note:* Population‐based registries, prospective observational cohorts, cooperative‐group datasets, transplant and cellular therapy registries, and administrative databases provide complementary information on treatment effectiveness, toxicity, treatment patterns, and long‐term outcomes in routine clinical practice. Their respective strengths and limitations should be considered when interpreting real‐world evidence.

Abbreviations: AE, adverse event; HF, heart failure; HTN, hypertension; NDTI, newly diagnosed transplant‐ineligible; RCT, randomized clinical trial; RWD, real‐world data.

Transplant and cellular therapy registries, including large international databases, provide robust information on outcomes following autologous transplantation and, more recently, CAR‐T cell therapy in routine practice. These datasets are particularly important because pivotal CAR‐T trials enrolled highly selected patients with adequate performance status, organ function, and limited comorbidity, whereas real‐world registries capture broader populations treated after regulatory approval [34, 35, 52, 58]. They are essential for understanding how intensive strategies perform outside trials, including in somewhat older populations. However, by design, they include only patients selected for these procedures and therefore do not capture the broader MM population deemed ineligible.

The rapid introduction of bispecific antibodies has created a similar need for high‐quality RWE. Although pivotal trials demonstrated substantial activity of BCMA‐ and GPRC5D‐directed bispecific antibodies, real‐world experience is required to define their effectiveness, infection risk, cytokine release syndrome management, treatment persistence, and use in patients with advanced age, frailty, or multiple comorbidities who are often under‐represented in registration studies [40, 41, 61, 62, 63, 64].

Administrative and claims‐based datasets offer very large sample sizes and long‐term follow‐up, particularly in older populations [59]. These data are valuable for studying treatment patterns, healthcare utilization, and survival at scale. Their main limitations include limited disease‐specific detail, potential misclassification, and lack of standardized response and toxicity assessments.

Across these diverse data sources, a consistent pattern emerges: real‐world MM populations are older, more heterogeneous, and more frequently affected by frailty and comorbidity than patients enrolled in RCTs. These differences influence treatment selection, dose intensity, toxicity, and treatment persistence, thereby providing essential context for interpreting the gap between trial efficacy and real‐world effectiveness [10, 11, 12, 43, 44, 45, 46, 50, 51, 52] At the same time, the interpretability of RWD remains constrained by important methodological limitations. The absence of standardized endpoints, inconsistent response assessment, variable data quality, and the limited integration of formal frailty measures complicate direct comparisons with RCT outcomes and may introduce substantial bias [13, 14, 15, 16, 17, 18, 19, 20, 21, 22, 23, 24, 25, 26, 27, 28]. Without careful design and analysis, RWD risk reflects patterns of care rather than true treatment effects. Approaches such as propensity‐score methods, target‐trial emulation, time‐dependent analyses, and other causal‐inference frameworks are increasingly required to reduce confounding and improve the interpretability of observational comparisons.

Despite these limitations, RWD are indispensable for understanding how MM therapies perform in the patients most commonly encountered in clinical practice. Their greatest value lies not in replacing RCTs but in contextualizing their results—defining the boundaries within which trial‐derived evidence can be safely and meaningfully applied.

## 4 Efficacy Gaps: Response, Progression‐Free Survival, and Overall Survival

A central question in reconciling RCTs and RWD in MM is whether the efficacy observed under controlled conditions—high response rates, deep remissions, and prolonged progression‐free and overall survival—is reproducible in routine clinical practice. Across disease settings, the answer is conditional: RCTs provide reliable estimates of comparative efficacy and relative treatment benefit, whereas the absolute outcomes achieved in routine practice are strongly influenced by patient characteristics, treatment exposure, and competing risks. Thus, outcomes observed in RCTs are most reproducible in patients who resemble trial populations but are frequently attenuated in older, frail, and comorbid patients who dominate real‐world cohorts [4, 5, 6, 7, 9, 29, 30, 31, 32, 33, 34, 35, 36, 37, 38, 46, 50, 51, 52, 57, 65, 66].

In NDTE disease, landmark trials have demonstrated that modern induction strategies followed by autologous stem cell transplantation and maintenance can achieve sustained disease control in fit patients, with progression‐free survival (PFS) extending beyond 5 years in some series [4, 5, 6, 7]. Real‐world registry data suggest that similar outcomes can be achieved when these approaches are applied to patients with comparable characteristics—typically younger, with preserved organ function and limited comorbidity [35, 36, 52, 58]. However, when these strategies are extended to older or more clinically complex patients, real‐world studies consistently report shorter progression‐free survival, higher rates of treatment‐related complications, and increased early mortality, reflecting both disease biology and host vulnerability, as well as reduced treatment intensity and limited treatment persistence [35, 52].

In the transplant‐ineligible setting, RCTs have established the efficacy of continuous, combination‐based regimens, including monoclonal antibody–containing approaches, with substantial gains in progression‐free and overall survival compared with historical standards [9, 29, 30, 31]. In selected elderly patients, real‐world outcomes may approximate those observed in trials [32, 46, 51, 52]. Conversely, population‐based cohorts including very elderly and frail individuals frequently demonstrate lower response rates, shorter survival, and higher rates of early treatment discontinuation, highlighting the impact of frailty, competing risks, and limited treatment tolerance [10, 11, 12, 43, 44, 45, 46]. In these patients, therapeutic benefit is constrained not only by disease aggressiveness but also by limited treatment tolerance and competing risks of morbidity and mortality.

A similar pattern is observed in relapsed/refractory MM. This issue has become particularly relevant with the introduction of cellular therapies and bispecific antibodies, where pivotal studies enrolled highly selected patients and post‐approval real‐world cohorts are increasingly required to define effectiveness, toxicity, treatment persistence, and outcomes in broader populations.

Although pivotal trials of triplet regimens and novel agents have demonstrated significant improvements in progression‐free and overall survival, real‐world analyses consistently show that patients treated outside trials are older, more heavily pretreated, and more comorbid [33, 34, 35, 36, 37, 38, 46, 50, 51, 52, 57, 65, 66]. In these populations, response rates and durability of benefit are often reduced, particularly in those with advanced organ dysfunction or highly refractory disease. Nevertheless, when real‐world patients approximate trial eligibility criteria, outcomes are frequently comparable, reinforcing the external validity of RCTs within that subset [32, 46, 50, 51, 52, 57, 65, 66].

These observations suggest that the divergence between RCT and real‐world outcomes is not a failure of trial validity but a predictable consequence of differences in patient selection, treatment exposure, and competing risks. Biologically and clinically, real‐world populations may present with more advanced disease, adverse cytogenetic features, cumulative treatment‐related damage, and greater clonal complexity, particularly in later lines of therapy [50, 51, 52]. Clinically, higher comorbidity burden and frailty lead to more conservative initial dosing, more frequent dose reductions and delays, and shorter treatment duration, all of which attenuate cumulative treatment intensity [32, 46, 50, 51, 52]. Methodologically, outcome assessment in routine practice is less standardized, with less frequent monitoring and incomplete documentation, potentially leading to underestimation of response depth and progression‐free survival [10, 43, 44, 45, 46, 59].

In this context, real‐world treatment outcomes in MM are jointly determined by the intrinsic antimyeloma activity of a regimen, its toxicity profile, and the ability to maintain therapy over time. RCTs define the efficacy of a regimen under optimal conditions, whereas RWD capture how toxicity, dose modifications, and treatment persistence modify this efficacy in routine practice. Failure to account for these dimensions may lead to unrealistic expectations regarding the absolute benefit achievable when modern therapies are applied to unselected patient populations.

Taken together, RCT and real‐world evidence should be viewed as complementary rather than discordant. RCTs establish the comparative efficacy and potential benefit of therapies under controlled conditions, whereas RWD defines the effectiveness achieved across the broader clinical spectrum of MM. Integrating these perspectives is essential to derive realistic expectations of benefit and to guide treatment decisions in the heterogeneous populations encountered in everyday practice.

## 5 Toxicity, Dose‐Intensity, and Treatment Discontinuation in the Real‐World

If differences in efficacy between randomized clinical trials (RCTs) and real‐world data (RWD) are often explainable by patient selection and disease biology, discrepancies in toxicity, dose intensity, and treatment persistence represent a more direct and clinically consequential source of divergence in treatment outcomes. In routine practice, these factors critically shape the cumulative effectiveness of therapy and are a major driver of the gap between trial efficacy and real‐world effectiveness [32, 43, 44, 45, 46, 50, 51, 52, 57, 65, 66].

RCTs are conducted under tightly controlled conditions, with predefined dose‐modification algorithms, intensive monitoring, and proactive management of adverse events. Importantly, eligibility criteria select for patients with preserved organ function, limited comorbidity burden, and good performance status, thereby reducing baseline susceptibility to severe toxicity [4, 5, 6, 7, 8, 9, 29, 30, 31, 32, 33, 34, 35, 36, 37, 38]. Even so, modern MM regimens are associated with clinically relevant toxicity profiles in trials, including hematologic toxicity, infections, cardiovascular events, neuropathy, and treatment‐related complications requiring dose adjustment or temporary interruption [30, 31, 33, 34, 35, 36, 37].

In real‐world practice, these toxicities are amplified by patient heterogeneity and physiological vulnerability. Older age, multimorbidity, frailty, and polypharmacy increase susceptibility to adverse events and reduce physiological reserve [10, 11, 12, 43, 44, 45, 46]. As a result, clinicians frequently initiate therapy at reduced doses compared with trial protocols, particularly for proteasome inhibitors, immunomodulatory agents, and corticosteroids [32, 46, 50, 51, 52]. Dose reductions, treatment delays, and temporary interruptions occur earlier and more frequently than in RCTs, reflecting the combined effects of treatment‐related toxicity, frailty, comorbidity, and reduced physiological reserve [32, 46, 50, 51, 52, 57, 65, 66].

These modifications have a direct impact on treatment persistence, which represents a key determinant of real‐world effectiveness. Early unplanned discontinuation due to toxicity, functional decline, or patient preference is substantially more common than reported in clinical trials [32, 46, 50, 51, 52, 57, 65, 66]. In frail and very elderly patients, discontinuation may occur after only a few cycles, effectively truncating exposure to active therapy and limiting the achievement of deep or durable responses. This phenomenon is particularly relevant for continuous‐treatment strategies, in which sustained treatment exposure is a prerequisite for achieving the full therapeutic benefit demonstrated in randomized trials.

Representative comparisons between pivotal randomized clinical trials and corresponding real‐world cohorts are summarized in Table 3, illustrating how differences in patient selection, treatment exposure, toxicity, and treatment persistence contribute to the gap between trial efficacy and real‐world effectiveness.

**TABLE 3:** Translation of pivotal randomized clinical trial results into routine clinical practice: Representative comparisons between randomized trials and corresponding real‐world cohorts.

Therapeutic regimen	Pivotal randomized clinical trial	Corresponding real‐world evidence	Trial efficacy (RCT)	Findings in routine clinical practice	Main factors explaining differences
Carfilzomib‐Rd	ASPIRE [35]	Large multicenter RWE cohorts [55, 56]	Significant improvement in ORR, PFS and OS in fit RRMM patients	Clinical benefit confirmed, but lower treatment persistence and more frequent dose reductions; cardiovascular toxicity more common in older and comorbid patients	Older age, cardiovascular comorbidity, dose attenuation, early discontinuation
Daratumumab‐Rd	MAIA [31]	Multicenter real‐world cohorts of transplant‐ineligible patients [18, 31, 45, 51]	Marked improvement in PFS and depth of response	Similar benefit in fit elderly patients, but attenuated effectiveness in frail populations owing to treatment interruptions and infections	Frailty, infections, reduced lenalidomide/steroid exposure, treatment discontinuation
Daratumumab‐VMP	ALCYONE [30]	Population‐based elderly cohorts [18, 31]	Superior PFS and OS compared with VMP	Benefit maintained but reduced in very elderly patients; lower delivered dose intensity and higher infection burden	Frailty, comorbidity, reduced chemotherapy dose intensity
Lenalidomide‐dexamethasone	FIRST [9]	Population‐based registries and observational cohorts [10, 11, 12, 42, 43, 44, 45]	Continuous Rd. superior to MPT with durable disease control	Higher rates of dose reduction, functional decline and early discontinuation; shorter PFS in frail patients	Frailty, competing mortality, reduced treatment persistence
BCMA‐directed CAR‐T cells (ide‐cel, cilta‐cel)	KarMMa [39]; CARTITUDE‐1 [40]	EBMT and CIBMTR registries [21, 22, 39]	Very high response rates and durable remissions in selected triple‐class exposed RRMM	Early real‐world studies confirm substantial activity, but broader patient selection, manufacturing delays, prolonged cytopenias and infections influence outcomes	Patient selection, access to therapy, toxicity, supportive care
Bispecific antibodies (teclistamab, talquetamab) [40, 41]	MajesTEC‐1; MonumenTAL‐1	Early multicenter post‐approval real‐world studies	High response rates in heavily pretreated RRMM	Clinical activity confirmed, although higher infection rates, prolonged immunosuppression and treatment interruptions are frequently reported	Broader eligibility, infection risk, longer follow‐up needed

*Note:* Representative comparisons between pivotal randomized clinical trials (RCTs) and corresponding real‐world evidence (RWE) studies across major therapeutic classes in multiple myeloma. While RWE generally confirms the relative treatment benefit observed in randomized trials, absolute clinical effectiveness is frequently attenuated in routine practice because of greater patient frailty, comorbidity burden, reduced dose intensity, and earlier treatment discontinuation. Emerging real‐world studies of CAR‐T cells and bispecific antibodies are extending these observations to contemporary immunotherapies.

Importantly, toxicity in real‐world MM is not merely an adverse event profile but a mechanistic driver of reduced effectiveness. Dose reductions and interruptions decrease delivered dose intensity, while early discontinuation shortens treatment exposure, thereby attenuating the cumulative biological impact of therapy. In this sense, toxicity and persistence act as mediators between RCT‐defined efficacy and real‐world outcomes, directly shaping the degree to which trial benefits can be translated into routine practice. Importantly, toxicity in real‐world MM is not merely an adverse event profile but a mechanistic driver of reduced effectiveness. Dose reductions and interruptions decrease delivered dose intensity, while early discontinuation shortens treatment exposure, thereby attenuating the cumulative biological impact of therapy. In this sense, toxicity and persistence act as mediators between RCT‐defined efficacy and real‐world outcomes, directly shaping the degree to which trial benefits can be translated into routine practice.

These observations reinforce a central principle: In MM, real‐world benefit depends not only on the intrinsic efficacy of a regimen but also on the ability to maintain adequate dose intensity and treatment duration in individual patients. Toxicity and treatment discontinuation therefore function as key mediators linking trial‐defined efficacy with real‐world effectiveness.

The conceptual framework linking patient selection, treatment delivery, toxicity, dose intensity, treatment persistence, and clinical outcomes is illustrated in Figure 1.

**FIGURE 1:**
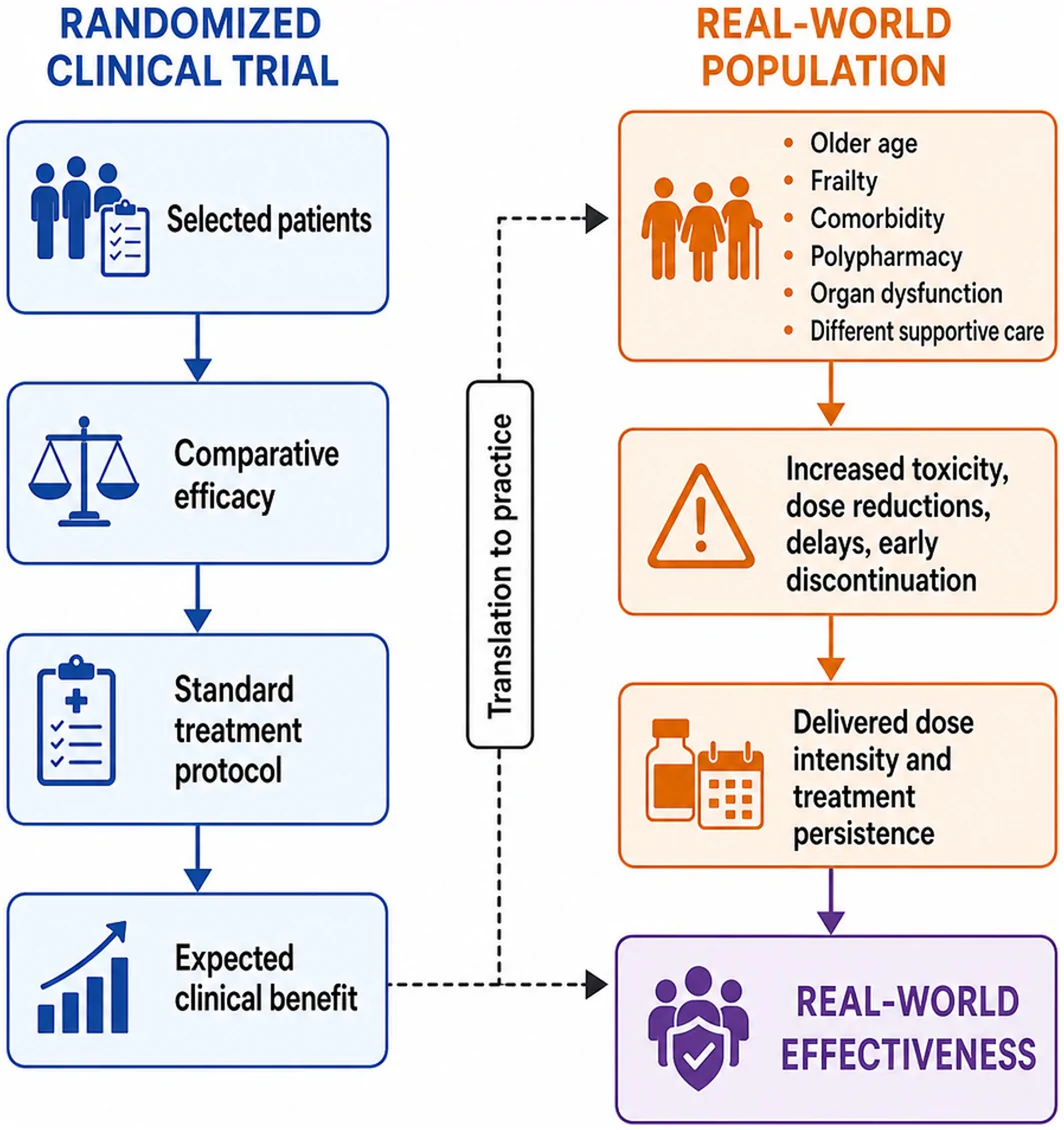
From randomized trial efficacy to real‐world effectiveness in multiple myeloma. Schematic representation of the mechanisms underlying the efficacy–effectiveness gap in multiple myeloma. Randomized clinical trials (RCTs) evaluate therapeutic efficacy in selected patients treated under controlled conditions, whereas real‐world populations are older, more heterogeneous, and more frequently affected by frailty, comorbidities, and organ dysfunction. These patient‐related factors increase the risk of treatment‐related toxicity, leading to dose reductions, treatment delays, and early discontinuation, which reduce delivered dose intensity and treatment persistence. Consequently, although the relative treatment benefit observed in RCTs may be preserved, the absolute clinical effectiveness achieved in routine practice is frequently attenuated. The figure illustrates how patient characteristics and treatment delivery mediate the translation of trial efficacy into real‐world outcomes.

## 6 Frailty, Comorbidities, and Geriatric Assessment: The Missing Link

The divergence between RCT efficacy and real‐world effectiveness in MM is ultimately mediated by patient‐level factors, among which frailty and comorbidity play a central role. While chronological age is often used as a surrogate for treatment tolerance, it is an imprecise marker of physiological reserve. Patients of similar age may differ substantially in functional status, comorbidity burden, cognitive capacity, and social support—dimensions that directly influence both the feasibility and the outcomes of therapy.

Frailty provides a more clinically meaningful framework to capture this heterogeneity than chronological age alone. In MM, disease‐specific frailty assessment tools, most notably the International Myeloma Working Group (IMWG) frailty index, have demonstrated that frailty independently predicts treatment‐related toxicity, early treatment discontinuation, and survival [47]. Patients classified as frail experience higher rates of severe adverse events, reduced treatment exposure, and inferior outcomes compared with fit patients, even when treated with the same regimens and in the absence of major differences in disease biology [47, 48, 49]. These observations position frailty as an important determinant of the gap between trial efficacy and real‐world effectiveness. Despite this evidence, frailty remains insufficiently integrated into the design and interpretation of RCTs. Eligibility criteria continue to rely predominantly on age thresholds, performance status, and basic organ function parameters, which fail to capture the multidimensional nature of vulnerability in older patients [4, 5, 6, 7, 8, 9, 29, 30, 33, 34, 35, 36, 37, 38]. As a result, trial populations are enriched for physiologically robust individuals, and the outcomes reported may overestimate the benefit achievable in frail patients. The absence of standardized frailty assessment in most pivotal trials represents a major limitation of current evidence generation and contributes to the limited external validity of RCT findings.

RWD, in contrast, more closely capture the heterogeneity of frailty observed in the MM population, particularly among older patients who are frequently under‐represented in clinical trials [10, 11, 12, 43, 44, 45, 46]. Observational studies incorporating frailty measures consistently show that frailty influences both clinical outcomes and treatment delivery, as clinicians frequently modify regimen intensity, dose, and supportive care according to perceived vulnerability [47, 48, 49]. However, frailty assessment in RWD remains heterogeneous and incompletely standardized. Different cohorts apply different instruments, including MM‐specific frailty scores, comorbidity indices, functional measures, or performance status‐based surrogates, while some datasets lack formal frailty evaluation altogether. This variability limits direct comparisons across studies and complicates the incorporation of frailty into comparative effectiveness analyses.

Several frailty assessment tools have been developed and validated in MM, differing in complexity, domains assessed, and feasibility of implementation. The International Myeloma Working Group (IMWG) frailty index represents the most widely validated MM‐specific instrument and integrates age, comorbidity, functional status, and performance status. Simplified approaches and comprehensive geriatric assessments provide complementary strategies depending on the clinical setting and available data sources (Table 4) [47, 48, 49].

**TABLE 4:** Frailty assessment tools in multiple Myeloma and their applications in randomized clinical trials and real‐world studies.

Frailty assessment tool	Domains assessed	Key features	Application in randomized clinical trials (RCTs)	Application in real‐world data (RWD) studies
International Myeloma Working Group (IMWG) Frailty Index [47]	Age, Charlson Comorbidity Index, Activities of Daily Living (ADL), Instrumental Activities of Daily Living (IADL), ECOG performance status	MM‐specific, validated, classifies patients as fit/intermediate‐fit/frail; strongest prognostic evidence in MM	Stratification factor; predefined subgroup analyses; treatment adaptation according to frailty status	Standardized risk adjustment; outcome stratification; comparative effectiveness analyses
Revised IMWG Frailty Score [48]	Age, ECOG performance status, Charlson Comorbidity Index	Simplified version of IMWG index; more feasible in routine practice	Pragmatic trial stratification; eligibility refinement in elderly‐focused studies	Broad implementation in registry‐based studies and electronic health record datasets
**Simplified Frailty Models (age–ECOG–CCI based)** [48]	Age, ECOG performance status, comorbidity burden	Rapid bedside tools; limited granularity but high feasibility	Exploratory stratification in pragmatic trials	Large‐scale application in observational cohorts; minimal data requirement
Comprehensive Geriatric Assessment (CGA)	functional status, cognition, nutrition, comorbidity, polypharmacy, social support	Multidimensional “gold standard” geriatric evaluation	Treatment individualization in elderly‐specific trials; adaptive dosing strategies	Detailed vulnerability profiling; supportive care optimization; treatment decision support
Myeloma‐Specific Frailty Adaptations (Mixed Models)	Functional reserve, comorbidity burden, treatment vulnerability proxies	Heterogeneous approaches combining clinical and functional parameters	Exploratory endpoints in early‐phase or adaptive trial designs	Real‐world prediction of toxicity, treatment discontinuation, and survival outcomes

*Note:* Frailty assessment tools differ in the domains evaluated, complexity, and feasibility of implementation. The International Myeloma Working Group frailty index represents the most widely validated MM‐specific instrument, whereas simplified scores and geriatric assessments provide complementary approaches for clinical practice, randomized trials, and real‐world studies.

From a mechanistic perspective, frailty can be understood as the principal factor modulating the relationship between treatment exposure and outcome. Frail patients are more likely to experience toxicity, undergo dose reductions, and discontinue therapy early, thereby reducing both treatment intensity and duration. In this way, frailty directly influences the tolerability and persistence components of treatment effectiveness, linking the biological efficacy of a regimen to its real‐world impact. Accordingly, frailty primarily influences the absolute effectiveness achieved in routine practice, whereas the relative treatment benefit observed in randomized comparisons may remain largely preserved. Without accounting for frailty, the interpretation of both RCT and RWD remains incomplete.

These considerations underscore the need to move beyond age‐based treatment paradigms toward frailty‐informed decision‐making. Future randomized trials should routinely incorporate validated frailty instruments as stratification factors, predefined subgroup variables, and determinants of treatment adaptation. Likewise, observational studies should adopt standardized frailty assessments to improve comparability across cohorts and strengthen comparative‐effectiveness analyses.

A more consistent and standardized incorporation of frailty assessment into both randomized clinical trials and observational cohorts is required to improve risk stratification, treatment individualization, and the external validity of multiple myeloma research. From a practical perspective, treatment intensity, dosing, and scheduling should be adapted according to physiological reserve rather than chronological age alone. In selected frail patients, early dose attenuation and individualized treatment schedules may improve tolerability, preserve treatment persistence, and ultimately maximize real‐world effectiveness.

## 7 Practical Synthesis: Translating Trial Evidence Into Patient‐Centered Treatment Decisions

Translating RCT evidence into routine clinical decisions requires integration of trial‐defined efficacy with real‐world estimates of treatment tolerability, dose intensity, and treatment persistence. RCTs provide the reference estimate of comparative treatment benefit under controlled conditions, whereas RWD inform the magnitude of benefit that can realistically be achieved in patients with different levels of frailty, comorbidity, and treatment vulnerability [4, 5, 6, 7, 8, 9, 29, 30, 31, 32, 33, 34, 35, 36, 37, 38]. The proposed approach consists of sequential steps: Identification of the evidence‐based regimen, assessment of patient similarity to trial populations, integration of real‐world effectiveness data, treatment adaptation, and continuous reassessment.

The second step is to determine how closely the individual patient resembles the population enrolled in the relevant clinical trial. This assessment should extend beyond chronological age and include performance status, comorbidity burden, organ function, prior therapies, and frailty as measured by validated instruments such as the IMWG frailty index [47]. This evaluation helps estimate whether trial‐derived efficacy estimates are likely to be achievable in the individual patient.

The third step involves explicit integration of relevant real‐world evidence. When available, observational studies and registry analyses provide estimates of treatment effectiveness, toxicity, and discontinuation rates in patient populations that more closely resemble those encountered in practice [10, 11, 12, 32, 46, 50, 51, 52, 57, 65, 66]. These data allow clinicians to refine expectations regarding absolute benefit and toxicity risk, particularly in older, frail, or comorbid patients, who are under‐represented in RCTs.

The fourth step is treatment adaptation according to the patient's biological reserve and anticipated ability to maintain therapy. In patients who closely resemble trial populations, standard dosing and schedules can generally be maintained. Conversely, in frail or highly comorbid patients, dose attenuation, schedule modification for example less frequent proteasome inhibitor administration, corticosteroid reduction, or selection of less intensive regimens may preserve treatment continuity, and reduce toxicity [32, 46, 50, 51, 52] Importantly, these adaptations should not be viewed as deviations from evidence‐based care but as necessary adjustments to preserve treatment persistence and overall treatment benefit in routine practice.

Supportive care represents an essential component of treatment adaptation. Infection prophylaxis, thrombosis prevention, cardiovascular monitoring, bone‐directed therapy, and interventions addressing nutrition and functional status are particularly important in vulnerable patients to maintain treatment continuity [10, 11, 12, 43, 44, 45, 46].

Finally, treatment decisions should remain dynamic. Early treatment cycles provide essential information regarding tolerability, functional decline, adherence, and patient preferences. Therapy should therefore be continuously adjusted according to toxicity, response, and changing clinical circumstances, allowing escalation when tolerated and de‐escalation when necessary.

This framework reflects the principles of evidence‐based medicine, in which clinical decisions integrate best available research evidence with clinical expertise and patient‐specific factors [67]. Treatment benefit emerges from the interaction between regimen efficacy, tolerability, dose intensity, and treatment persistence, with frailty acting as a major determinant of how therapy should be delivered. Thus, RCTs define what a treatment can achieve, whereas RWD help estimate what can realistically be achieved in a specific patient.

Although RWD are essential for this process, their interpretation requires caution because residual confounding, selection bias, incomplete capture of frailty, and variability in treatment documentation may influence observed outcomes. Nevertheless, structured integration of RCT and RWD evidence provides a practical framework for delivering individualized and evidence‐based care across the heterogeneous MM population.

## 8 Methodologic Priorities for Future Multiple Myeloma Real‐World Research

Bridging the gap between RCT efficacy and real‐world effectiveness in MM requires methodological approaches capable of generating reliable and clinically interpretable RWD. Although RWD are increasingly used to complement RCTs and support regulatory and clinical decision‐making, their value depends on standardized data collection, appropriate analytical methods, and accurate characterization of patient vulnerability. Current limitations, including heterogeneous data sources, inconsistent endpoint definitions, and incomplete capture of frailty and treatment delivery parameters, restrict comparability between RWD and RCT findings [13, 14, 15, 16, 17, 18, 19, 20, 21, 22, 23, 24, 25, 26, 27, 28, 43, 44, 45, 46].

A first priority is the standardization of core data elements and clinical endpoints across real‐world datasets. Harmonized definitions of response, progression, toxicity, treatment discontinuation, dose intensity, and treatment duration are essential to enable meaningful comparisons across cohorts and with RCT populations [13, 14, 15, 16, 17, 18, 19, 20, 21, 22, 23, 24, 25, 26, 27, 28, 32, 46, 50, 51, 52, 60]. Because treatment exposure strongly influences effectiveness in routine practice, dose modifications and treatment persistence should be considered key outcomes rather than secondary variables.

A second central priority is the routine integration of frailty and geriatric assessment into both RCTs and RWD. Frailty represents a major determinant of treatment tolerance, dose intensity, and survival, yet remains inconsistently measured and reported [47, 48, 49]. Standardized incorporation of validated tools, including the IMWG frailty index, should become part of prospective registries and clinical trials. Frailty should be considered not only a prognostic variable but also a factor guiding treatment adaptation and subgroup interpretation.

Third, analytical approaches must evolve to address the inherent biases of observational data. Confounding by indication, selection bias, and informative censoring are pervasive in RWD and can substantially distort estimates of treatment effect [13, 14, 15, 16, 17, 18, 19, 20, 21, 22, 23, 24, 25, 26, 27, 68]. Robust causal‐inference methods—including propensity score–based approaches, inverse probability weighting, marginal structural models, and, where appropriate, instrumental variable analyses—should be systematically applied [13, 14, 15, 16, 17, 18, 19, 20, 21, 22, 23, 24, 25, 26, 27, 28]. Transparent reporting of assumptions, sensitivity analyses, and the limits of causal interpretation is essential to ensure that RWD inform, rather than mislead, clinical decision‐making. However, these approaches cannot fully compensate for unmeasured variables, emphasizing the importance of prospective data collection and transparent reporting of analytical assumptions.

A fourth priority is closer alignment RCT and RWD populations through more pragmatic study designs. Pragmatic clinical trials with broader eligibility criteria, including and greater inclusion of older patients and individuals with controlled comorbidities, should be encouraged whenever feasible [14, 15, 16]. When fully pragmatic designs are not possible, unnecessary exclusion criteria should be minimized to improve representativeness. Registry‐based randomized trials and trial–registry linkages represent complementary approaches that combine the internal validity of randomization with the clinical diversity of real‐world populations [14, 15, 16]. These models may be particularly valuable in MM, where therapeutic innovation is rapid and patient heterogeneity is substantial [69].

The increasing use of RWD in regulatory science further highlights the need for methodological rigor. Regulatory agencies, including the US Food and Drug Administration and the European Medicines Agency, increasingly recognize RWE as complementary evidence for post‐approval commitments, safety monitoring, and, in selected circumstances, evaluation of effectiveness in broader populations. However, reliance on RWE requires predefined data standards, transparent analytical strategies, and appropriate validation to ensure reliable interpretation [13, 14, 15, 16, 17, 18, 19, 20, 21, 22, 23, 24, 25, 26, 27, 28].

In MM, the increasing incorporation of measurable residual disease (MRD) endpoints into clinical trials and regulatory pathways introduces additional challenges [70, 71]. While MRD provides highly sensitive measures of disease control, its relationship with long‐term clinical benefit may be less predictable in heterogeneous real‐world populations, particularly when patients differ substantially from trial participants in age, frailty, competing risks, and treatment exposure. Future RWE strategies should therefore evaluate whether surrogate endpoints validated in selected trial populations retain the same clinical meaning in routine practice.

Finally, outcome assessment should extend beyond survival endpoints to include functional status, quality of life, treatment burden, and patient‐reported outcomes. These measures are particularly relevant in older and frail patients, in whom the balance between efficacy and tolerability determines the clinical value of therapy [10, 11, 12, 47, 48, 49].

Finally, data quality and transparency must be strengthened. Prospective data collection, standardized definitions, rigorous data curation, and external validation are essential to ensure reliability [13, 14, 15, 16, 17, 18, 19, 20, 21, 22, 23, 24, 25, 26, 27, 28]. Equally important is the clear communication of uncertainty, including the limitations of data sources and analytical methods. RWD should not be viewed as inherently inferior or superior to RCT evidence but as complementary, with its value determined by methodological rigor.

Taken together, these priorities define a roadmap for integrating RCTs and RWD into a unified evidence framework for MM. Standardized data collection, routine frailty assessment, robust causal methods, pragmatic study designs, and transparent reporting will allow generation of evidence that more accurately reflects the patients treated in everyday practice. The ultimate goal is not to replace RCTs but to ensure that their findings are interpreted within the broader clinical context in which therapies are delivered.

## 9 Conclusion

MM illustrates how differences in patient selection, treatment delivery, and follow‐up contribute to systematic divergence between randomized clinical trial (RCT) outcomes and real‐world effectiveness. RCTs have driven major therapeutic advances and define regimen efficacy under controlled conditions, but they predominantly include younger, fitter, and less comorbid patients than those encountered in routine practice [4, 5, 6, 7, 8, 9, 29, 30, 31, 32, 33, 34, 35, 36, 37, 38]. In contrast, real‐world populations are older, more heterogeneous, and frequently frail [1, 2, 3, 10, 11, 12, 43, 44, 45, 46], which contributes to differences between trial outcomes and routine clinical results.

In routine practice, outcomes are determined not only by intrinsic treatment efficacy but also by tolerability, dose intensity, and treatment persistence [32, 46, 50, 51, 52, 57, 65, 66]. RCTs primarily estimate the comparative efficacy of regimens, whereas real‐world data capture how toxicity, comorbidity, and treatment delivery modify these effects, resulting in differences in observed effectiveness.

Frailty represents a central mediator of this divergence. By integrating comorbidity burden, functional status, and physiological reserve, frailty better predicts toxicity, treatment discontinuation, and survival than chronological age alone [47, 48, 49]. However, its inconsistent assessment in both RCTs and real‐world studies limits its current clinical and methodological utility.

RCTs and real‐world data should be viewed as complementary evidence sources: RCTs define what therapies can achieve under ideal conditions, while real‐world data describe what is achieved across heterogeneous patient populations [13, 14, 15, 16, 17, 18, 19, 20, 21, 22, 23, 24, 25, 26, 27, 28]. Their integrated interpretation enables more accurate estimation of benefit–risk balance and supports more individualized treatment decisions.

Future progress in MM will depend on aligning evidence generation with clinical reality. Standardized real‐world datasets, routine incorporation of frailty assessment, robust causal inference methods, and more pragmatic trial designs are key methodological priorities [15, 16, 17, 18, 19, 20, 21, 22, 23, 24, 25, 26, 27, 28, 29, 30]. In parallel, more consistent reporting of treatment exposure, dose modifications, and discontinuation patterns will be essential to interpret therapeutic benefit in routine practice. Together, these strategies may help integrate RCT and real‐world evidence into a unified framework that better reflects the complexity of patients with MM and supports truly individualized care.

## Author Contributions

All authors contributed to the manuscript and were involved in revisions and proofreading. All authors approved the submitted version. Open access publishing facilitated by Universita della Calabria, as part of the Wiley ‐ CRUI‐CARE agreement.

## Funding

The authors have nothing to report.

## Ethics Statement

The authors have nothing to report.

## Conflicts of Interest

The authors declare no conflicts of interest.

## Data Availability

Data sharing not applicable to this article as no datasets were generated or analysed during the current study.

## References

[1] R. A. Kyle and S. V. Rajkumar , “Multiple Myeloma,” Blood 111, no. 6 (2008): 2962–2972, 10.1182/blood-2007-10-078022. PMC2265446 18332230

[2] S. Y. Kristinsson , O. Landgren , P. W. Dickman , A. R. Derolf , and M. Björkholm , “Patterns of Survival in Multiple Myeloma: A Population‐Based Study of Patients Diagnosed in Sweden From 1973 to 2003,” Journal of Clinical Oncology 25, no. 15 (2007): 1993–1999, 10.1200/JCO.2006.09.0100. 17420512

[3] N. Kahlon , N. Bansal , S. Baddam , J. Rajput , and Z. Qureshi , “Targeted Therapeutics and U.S. Population‐Level Mortality Trends in Multiple Myeloma: A SEER‐Based Analysis From 1975 to 2023,” Oncotarget 17, no. 1 (2026): 216–225, 10.18632/oncotarget.28877. PMC13249087 42047296

[4] M. Attal , V. Lauwers‐Cances , C. Hulin , et al., “Lenalidomide, Bortezomib, and Dexamethasone With Transplantation for Myeloma,” New England Journal of Medicine 376, no. 14 (2017): 1311–1320, 10.1056/NEJMoa1611750. PMC6201242 28379796

[5] P. G. Richardson , S. J. Jacobus , E. A. Weller , et al., “Triplet Therapy, Transplantation, and Maintenance Until Progression in Myeloma,” New England Journal of Medicine 387, no. 2 (2022): 132–147. 10.1056/NEJMoa2204925 PMC10040899 35660812

[6] P. Moreau , M. Attal , C. Hulin , et al., “Bortezomib, Thalidomide, and Dexamethasone With or Without Daratumumab Before and After Autologous Stem‐Cell Transplantation for Newly Diagnosed Multiple Myeloma (CASSIOPEIA): A Randomised, Open‐Label, Phase 3 Study,” Lancet 394, no. 10192 (2019): 29–38, 10.1016/S0140-6736(19)31240-1. 31171419

[7] P. M. Voorhees , J. L. Kaufman , J. Laubach , et al., “Daratumumab, Lenalidomide, Bortezomib, and Dexamethasone for Transplant‐Eligible Newly Diagnosed Multiple Myeloma: The GRIFFIN Trial,” Blood 136, no. 8 (2020): 936–945. 10.1182/blood.2020005288 PMC7441167 32325490

[8] R. Mina , P. Musto , D. Rota‐Scalabrini , et al., “Carfilzomib Induction, Consolidation, and Maintenance With or Without Autologous Stem‐Cell Transplantation in Patients With Newly Diagnosed Multiple Myeloma: Pre‐Planned Cytogenetic Subgroup Analysis of the Randomised, Phase 2 FORTE Trial,” Lancet Oncology 24, no. 1 (2023): 64–76. 10.1016/S1470-2045(22)00693-3 36528035

[9] L. Benboubker , M. A. Dimopoulos , A. Dispenzieri , et al., “Lenalidomide and Dexamethasone in Transplant‐Ineligible Patients With Myeloma,” New England Journal of Medicine 371, no. 10 (2014): 906–917, 10.1056/NEJMoa1402551. 25184863

[10] M. Engelhardt , G. Ihorst , J. Duque‐Afonso , et al., “Structured Assessment of Frailty in Multiple Myeloma as a Paradigm of Individualized Treatment Algorithms in Cancer Patients at Advanced Age,” Haematologica 105, no. 5 (2020): 1183–1188, 10.3324/haematol.2019.242958. PMC7193478 32241848

[11] K. Yong , M. Delforge , and C. Driessen , “Multiple Myeloma: Patient Outcomes in Real‐World Practice,” British Journal of Haematology 175, no. 2 (2016): 252–264, 10.1111/bjh.14213. PMC5096152 27411022

[12] T. Facon , X. Leleu , and S. Manier , “How I Treat Multiple Myeloma in Geriatric Patients,” Blood 143, no. 3 (2024): 224–232. 10.1182/blood.2022017635 PMC10808246 36693134

[13] R. E. Sherman , S. A. Anderson , G. J. Dal Pan , et al., “Real‐World Evidence—What Is It and What Can It Tell us?,” New England Journal of Medicine 375, no. 23 (2016): 2293–2297, 10.1056/NEJMsb1609216. 27959688

[14] H. Yuan , M. S. Ali , E. S. Brouwer , et al., “Real‐World Evidence: What It Is and What It Can Tell us According to the International Society for Pharmacoepidemiology (ISPE) Comparative Effectiveness Research (CER) Special Interest Group (SIG),” Clinical Pharmacology and Therapeutics 104 (2018): 239–241, 10.1002/cpt.1086. 29733448

[15] A. Makady , A. de Boer , and H. Hillege , “What Is Real‐World Data? A Review of Definitions Based on Literature and Stakeholder Interviews,” Value in Health 20, no. 7 (2017): 858–865, 10.1016/j.jval.2017.03.008. 28712614

[16] M. A. Hernán , I. J. Dahabreh , B. A. Dickerman , and S. A. Swanson , “The Target Trial Framework for Causal Inference From Observational Data: Why and When Is It Helpful?,” Annals of Internal Medicine 178, no. 3 (2025): 402–407, 10.7326/ANNALS-24-01871. PMC11936718 39961105

[17] B. Bermejo , R. Dienstmann , and M. Bellet , “Maximizing the Impact of Real‐World Evidence on Cancer Treatment: What Do We Need to Achieve It?,” Journal of Comparative Effectiveness Research 15 (2026): e250186. 10.57264/cer-2025-0186 PMC13154927 42029173

[18] E. A. Martino , C. Conticello , and E. Zamagni , “Carfilzomib Combined With Lenalidomide and Dexamethasone (KRd) as Salvage Therapy for Multiple Myeloma Patients: Italian, Multicenter, Retrospective Clinical Experience With 600 Cases Outside of Controlled Clinical Trials,” Hematological Oncology 40, no. 5 (2022): 1009–1019, 10.1002/hon.3035. 35638723

[19] A. Bruzzese , D. Derudas , M. Galli , and E. A. Martino , “Elotuzumab Plus Lenalidomide and Dexamethasone in Relapsed/Refractory Multiple Myeloma: Extended 3‐Year Follow‐Up of a Multicenter, Retrospective Clinical Experience With 319 Cases Outside of Controlled Clinical Trials,” Hematological Oncology 40, no. 4 (2022): 704–715. 10.1002/hon.3031 35608183

[20] E. A. Martino , S. Palmieri , and M. Galli , “Elotuzumab Plus Pomalidomide and Dexamethasone in Relapsed/Refractory Multiple Myeloma: Extended Follow‐Up of a Multicenter, Retrospective Real‐World Experience With 321 Cases Outside of Controlled Clinical Trials,” Hematological Oncology 42, no. 4 (2024): e3290. 10.1002/hon.3290 38818978

[21] M. Gentile , E. Vigna , and S. Palmieri , “Elotuzumab Plus Pomalidomide and Dexamethasone in Relapsed/Refractory Multiple Myeloma: A Multicenter, Retrospective, Real‐World Experience With 200 Cases Outside of Controlled Clinical Trials,” Haematologica 109, no. 1 (2024): 245–255. 10.3324/haematol.2023.283251 PMC10772491 37439329

[22] E. A. Martino , D. Derudas , and E. Rossi , “Efficacy and Prognostic Indicators of Isatuximab, Pomalidomide, and Dexamethasone (IsaPd) in Daratumumab‐Refractory Multiple Myeloma Patients: A Multicenter Real‐World Study,” Hematological Oncology 43, no. 2 (2025): e70042. 10.1002/hon.70042 PMC11789454 39898517

[23] E. A. Martino , D. Derudas , and E. Rossi , “Isatuximab, Pomalidomide, and Dexamethasone as Salvage Therapy for Patients With Multiple Myeloma: The Italian, Multicenter, Retrospective Clinical Experience With 270 Cases Outside of Controlled Clinical Trials,” Haematologica 110, no. 4 (2025): 1028–1033, 10.3324/haematol.2024.286658. PMC11959237 39605207

[24] E. A. Martino , A. Pitino , and M. Offidani , “Comparison of Isatuximab‐Pomalidomide‐Dexamethasone Versus Elotuzumab‐Pomalidomide‐Dexamethasone in Relapsed/Refractory Multiple Myeloma Patients: A Target Trial Emulation Using Real‐World Data,” Haematologica 110, no. 8 (2025): 1875–1881. 10.3324/haematol.2025.287325 PMC12358792 40761134

[25] M. Gentile , G. Specchia , and D. Derudas , “Elotuzumab, Lenalidomide, and Dexamethasone as Salvage Therapy for Patients With Multiple Myeloma: Italian, Multicenter, Retrospective Clinical Experience With 300 Cases Outside of Controlled Clinical Trials,” Haematologica 106, no. 1 (2021): 291–294. 10.3324/haematol.2019.241513 PMC7776255 32107338

[26] E. A. Martino , S. Palmieri , M. Galli , et al., “Outcomes and Prognostic Indicators in Daratumumab‐Refractory Multiple Myeloma: A Multicenter Real‐World Study of Elotuzumab, Pomalidomide, and Dexamethasone in 247 Patients,” ESMO Open 10, no. 2 (2025): 104084, 10.1016/j.esmoop.2024.104084. PMC11761902 39778329

[27] F. Morabito , E. Zamagni , and C. Conticello , “Survival Risk Scores for Real‐Life Relapsed/Refractory Multiple Myeloma Patients Receiving Elotuzumab or Carfilzomib in Combination With Lenalidomide and Dexamethasone as Salvage Therapy: Analysis of 919 Cases Outside Clinical Trials,” Frontiers in Oncology 12 (2022): 890376. 10.3389/fonc.2022.890376 PMC9341470 35924160

[28] F. Morabito , E. Zamagni , and C. Conticello , “Adjusted Comparison Between Elotuzumab and Carfilzomib in Combination With Lenalidomide and Dexamethasone as Salvage Therapy for Multiple Myeloma Patients,” European Journal of Haematology 108, no. 3 (2022): 178–189, 10.1111/ejh.13723. 34716957

[29] B. G. M. Durie , A. Hoering , M. H. Abidi , and S. V. Rajkumar , “Bortezomib With Lenalidomide and Dexamethasone Versus Lenalidomide and Dexamethasone Alone in Patients With Newly Diagnosed Myeloma Without Intent for Immediate Autologous Stem‐Cell Transplant (SWOG S0777): A Randomised, Open‐Label, Phase 3 Trial,” Lancet 389, no. 10068 (2017): 519–527. 10.1016/S0140-6736(16)31594-X PMC5546834 28017406

[30] M. V. Mateos , M. A. Dimopoulos , and M. Cavo , “Daratumumab Plus Bortezomib, Melphalan, and Prednisone for Untreated Myeloma,” New England Journal of Medicine 378, no. 6 (2018): 518–528, 10.1056/NEJMoa1714678. 29231133

[31] T. Facon , S. Kumar , T. Plesner , and R. Z. Orlowski , “Daratumumab Plus Lenalidomide and Dexamethasone for Untreated Myeloma,” New England Journal of Medicine 380, no. 22 (2019): 2104–2115, 10.1056/NEJMoa1817249. PMC10045721 31141632

[32] A. Larocca , F. Bonello , G. Gaidano , et al., “Dose/Schedule‐Adjusted Rd‐R vs Continuous Rd for Elderly, Intermediate‐Fit Patients With Newly Diagnosed Multiple Myeloma,” Blood 137, no. 22 (2021): 3027–3036, 10.1182/blood.2022018970. 33739404

[33] M. A. Dimopoulos , A. Oriol , H. Nahi , et al., “Daratumumab, Lenalidomide, and Dexamethasone for Multiple Myeloma,” New England Journal of Medicine 375, no. 14 (2016): 1319–1331, 10.1056/NEJMoa1607751. 27705267

[34] A. Palumbo , A. Chanan‐Khan , K. Weisel , et al., “Daratumumab, Bortezomib, and Dexamethasone for Multiple Myeloma,” New England Journal of Medicine 375, no. 8 (2016): 754–766, 10.1056/NEJMoa1606038. 27557302

[35] A. K. Stewart , S. V. Rajkumar , M. A. Dimopoulos , et al., “Carfilzomib, Lenalidomide, and Dexamethasone for Relapsed Multiple Myeloma,” New England Journal of Medicine 372, no. 2 (2015): 142–152, 10.1056/NEJMoa1411321. 25482145

[36] M. A. Dimopoulos , P. Moreau , A. Palumbo , et al., “Carfilzomib and Dexamethasone Versus Bortezomib and Dexamethasone for Patients With Relapsed or Refractory Multiple Myeloma (ENDEAVOR): A Randomised, Phase 3, Open‐Label, Multicentre Study,” Lancet Oncology 17, no. 1 (2016): 27–38, 10.1016/S1470-2045(15)00464-7. 26671818

[37] S. Lonial , M. Dimopoulos , A. Palumbo , et al., “Elotuzumab Therapy for Relapsed or Refractory Multiple Myeloma,” New England Journal of Medicine 373, no. 7 (2015): 621–631. 10.1056/NEJMoa1505654 26035255

[38] P. Moreau , T. Masszi , and N. Grzasko , “Oral Ixazomib, Lenalidomide, and Dexamethasone for Multiple Myeloma,” New England Journal of Medicine 374, no. 17 (2016): 1621–1634, 10.1056/NEJMoa1516282. 27119237

[39] N. C. Munshi , L. D. Anderson, Jr. , N. Shah , et al., “Idecabtagene Vicleucel in Relapsed and Refractory Multiple Myeloma,” New England Journal of Medicine 384, no. 8 (2021): 705–716, 10.1056/NEJMoa2024850. 33626253

[40] E. Hindié , “Teclistamab in Relapsed or Refractory Multiple Myeloma,” New England Journal of Medicine 387, no. 18 (2022): 1721–1723, 10.1056/NEJMc2211969. PMID: 36322859. 36322859

[41] A. Chari , M. C. Minnema , and J. G. Berdeja , “Talquetamab, a T‐Cell‐Redirecting GPRC5D Bispecific Antibody for Multiple Myeloma,” New England Journal of Medicine 387, no. 24 (2022): 2232–2244. 10.1056/NEJMoa2204591 36507686

[42] J. G. Berdeja , D. Madduri , and S. Z. Usmani , “Ciltacabtagene Autoleucel, a B‐Cell Maturation Antigen‐Directed Chimeric Antigen Receptor T‐Cell Therapy in Patients With Relapsed or Refractory Multiple Myeloma (CARTITUDE‐1): A Phase 1b/2 Open‐Label Study,” Lancet 398, no. 10297 (2021): 314–324. 10.1016/S0140-6736(21)00933-8 34175021

[43] I. Turesson , R. Velez , S. Y. Kristinsson , and O. Landgren , “Patterns of Improved Survival in Patients With Multiple Myeloma in the Twenty‐First Century: A Population‐Based Study,” Journal of Clinical Oncology 28, no. 5 (2010): 830–834. 10.1200/JCO.2009.25.4177 PMC2834396 20038719

[44] C. H. Blimark , A. J. Vangsted , T. W. Klausen , et al., “Outcome Data From >10 000 Multiple Myeloma Patients in the Danish and Swedish National Registries,” European Journal of Haematology 108, no. 2 (2022): 99–108. 10.1111/ejh.13707 34514635

[45] S. Y. Kristinsson , W. F. Anderson , and O. Landgren , “Improved Long‐Term Survival in Multiple Myeloma up to the Age of 80 Years,” Leukemia 28, no. 6 (2014): 1346–1348, 10.1038/leu.2014.23. 24418994

[46] A. Seesaghur , N. Petruski‐Ivleva , V. Banks , et al., “Real‐World Reproducibility Study Characterizing Patients Newly Diagnosed With Multiple Myeloma Using Clinical Practice Research Datalink, a UK‐Based Electronic Health Records Database,” Pharmacoepidemiology and Drug Safety 30, no. 2 (2021): 248–256, 10.1002/pds.5171. PMC7984077 33174338

[47] A. Palumbo , S. Bringhen , and M. V. Mateos , “Geriatric Assessment Predicts Survival and Toxicities in Elderly Myeloma Patients: An International Myeloma Working Group Report,” Blood 125, no. 13 (2015): 2068–2074, 10.1182/blood-2014-12-615187. PMC4375104 25628469

[48] M. D'Agostino , A. Larocca , M. Offidani , and A. M. Liberati , “Octogenarian Newly Diagnosed Multiple Myeloma Patients Without Geriatric Impairments: The Role of Age >80 in the IMWG Frailty Score,” Blood Cancer Journal 11, no. 4 (2021): 73. 10.1038/s41408-021-00464-w PMC8041817 33846296

[49] F. Smits , K. Groen , M. D. Levin , and C. A. M. Stege , “Frail Subgroups Determine Heterogeneous Outcomes in Older Patients With NDMM: Long‐Term Follow‐Up of the HOVON 143 Trial,” Blood Advances 9, no. 22 (2025): 5828–5836. 10.1182/bloodadvances.2025017394 PMC12663016 40845265

[50] G. H. Jackson , F. E. Davies , C. Pawlyn , and D. A. Cairns , “Lenalidomide Maintenance Versus Observation for Patients With Newly Diagnosed Multiple Myeloma (Myeloma XI): A Multicentre, Open‐Label, Randomised, Phase 3 Trial,” Lancet Oncology 20, no. 1 (2019): 57–73. 10.1016/S1470-2045(18)30687-9 PMC6318225 30559051

[51] A. Panopoulou , D. A. Cairns , A. Holroyd , and I. Nichols , “Optimizing the Value of Lenalidomide Maintenance by Extended Genetic Profiling: An Analysis of 556 Patients in the Myeloma XI Trial,” Blood 141, no. 14 (2023): 1666–1674. 10.1182/blood.2022018339 PMC10113174 36564045

[52] E. K. O'Donnell , J. P. Laubach , A. J. Yee , et al., “A Phase 2 Study of Modified Lenalidomide, Bortezomib and Dexamethasone in Transplant‐Ineligible Multiple Myeloma,” British Journal of Haematology 182, no. 2 (2018): 222–230, 10.1111/bjh.15261. PMC6074026 29740809

[53] S. Ailawadhi , S. Jagannath , M. Narang , et al., “Connect MM Registry as a National Reference for United States Multiple Myeloma Patients,” Cancer Medicine 9, no. 1 (2020): 35–42. 10.1002/cam4.2656 PMC6943143 31701679

[54] C. Costello , F. E. Davies , G. Cook , et al., “INSIGHT MM: A Large, Global, Prospective, Non‐Interventional, Real‐World Study of Patients With Multiple Myeloma,” Future Oncology 15, no. 13 (2019): 1411–1428, 10.2217/fon-2019-0013. PMC6854441 30816809

[55] K. Ramasamy , H. Goldschmidt , P. Moreau , et al., “P945: Real‐World Clinical Outcomes Among Triple‐Class Exposed Relapsed Refractory Multiple Myeloma Patients in US and Europe: A PREAMBLE Registry Study,” Hema 7 (2023): e642555a, 10.1097/01.HS9.0000970684.64255.5a.

[56] K. Ramasamy , R. Vij , D. Kuter , et al., “Real‐World Treatment Patterns and Clinical Outcomes in Patients With Multiple Myeloma Previously Treated With Lenalidomide and an Anti‐CD38 Monoclonal Antibody,” Clinical Lymphoma, Myeloma & Leukemia 25, no. 5 (2025): 337–348, 10.1016/j.clml.2024.12.002. 39799046

[57] B. Dhakal , J. He , J. M. Schecter , et al., “Real‐World Treatment Patterns and Outcomes in Patients With Lenalidomide‐Refractory Multiple Myeloma With 1 to 3 Prior Lines: SEER‐Medicare Database,” Clinical Lymphoma, Myeloma & Leukemia 25, no. 6 (2025): e394–e403. 10.1016/j.clml.2025.01.009 39966019

[58] S. Iacobelli , S. Schönland , L. Koster , et al., “Outcomes Following Different Upfront Stem Cell Transplantation Strategies for Multiple Myeloma: A Statistical Perspective on Behalf of the Chronic Malignancies Working Party of the EBMT,” Bone Marrow Transplantation 60, no. 10 (2025): 1361–1368, 10.1038/s41409-025-02675-2. PMC12568642 40739129

[59] L. Garderet , L. Gras , and L. Koster , “Global Characteristics and Outcomes of Autologous Hematopoietic Stem Cell Transplantation for Newly Diagnosed Multiple Myeloma: A Study of the Worldwide Network for Blood and Marrow Transplantation (WBMT),” American Journal of Hematology 99, no. 11 (2024): 2084–2095. 10.1002/ajh.27451 PMC11758675 39158218

[60] B. Dhakal , H. Einsele , J. M. Schecter , et al., “Real‐World Treatment Patterns and Outcomes in Relapsed/Refractory Multiple Myeloma (1‐3 Prior Lines): Flatiron Database,” Blood Advances 8, no. 19 (2024): 5062–5071. 10.1182/bloodadvances.2024012640 PMC11459902 39110988

[61] E. A. Martino , A. Bruzzese , C. Labanca , et al., “Teclistamab‐cqyv in Multiple Myeloma,” European Journal of Haematology 112, no. 3 (2024): 320–327. 10.1111/ejh.14121 37848191

[62] A. M. Lesokhin , M. H. Tomasson , B. Arnulf , et al., “Elranatamab in Relapsed or Refractory Multiple Myeloma: Phase 2 MagnetisMM‐3 Trial Results,” Nature Medicine 29, no. 9 (2023): 2259–2267, 10.1038/s41591-023-02528-9. PMC10504075 37582952

[63] M. E. Alvaro , E. A. Martino , S. Caserta , et al., “Elranatamab in Relapsed/Refractory Multiple Myeloma: Mechanisms, Clinical Evidence, and Emerging Perspectives,” European Journal of Haematology 117, no. 1 (2026): 42–54. 10.1111/ejh.70167 41846541

[64] C. Labanca , E. A. Martino , E. Vigna , et al., “Talquetamab in Multiple Myeloma: Efficacy, Safety, and Future Directions,” European Journal of Haematology 114, no. 3 (2025): 386–399. 10.1111/ejh.14353 PMC11798766 39604778

[65] S. Dong , R. Banerjee , A. M. Khan , et al., “Carfilzomib Prescribing Patterns and Outcomes for Relapsed or Refractory Multiple Myeloma: A Real‐World Analysis,” Blood Cancer Journal 15, no. 1 (2025): 48. 10.1038/s41408-025-01256-2 PMC11953251 40155649

[66] S. Atrash , A. Tullos , S. Panozzo , et al., “Cardiac Complications in Relapsed and Refractory Multiple Myeloma Patients Treated With Carfilzomib,” Blood Cancer Journal 5, no. 1 (2015): e272. 10.1038/bcj.2014.93 PMC4314456 25594159

[67] Evidence‐Based Medicine Working Group , “Evidence‐Based Medicine. A New Approach to Teaching the Practice of Medicine,” Journal of the American Medical Association 268, no. 17 (1992): 2420–2425. 10.1001/jama.1992.03490170092032 1404801

[68] M. A. Hernán and J. M. Robins , “Using Big Data to Emulate a Target Trial When a Randomized Trial Is Not Available,” American Journal of Epidemiology 183, no. 8 (2016): 758–764. 10.1093/aje/kwv254 PMC4832051 26994063

[69] M. S. Lauer and R. B. D'Agostino, Sr. , “The Randomized Registry Trial—The Next Disruptive Technology in Clinical Research?,” New England Journal of Medicine 369, no. 17 (2013): 1579–1581. 10.1056/NEJMp1310102 23991657

[70] O. Landgren , T. J. Prior , T. Masterson , et al., “EVIDENCE Meta‐Analysis: Evaluating Minimal Residual Disease as an Intermediate Clinical End Point for Multiple Myeloma,” Blood 144, no. 4 (2024): 359–367, 10.1182/blood.2024024371. PMC11418064 38768337

[71] B. A. Derman and R. Fonseca , “Measurable Residual Disease and Decision‐Making in Multiple Myeloma,” Hematology/Oncology Clinics of North America 38, no. 2 (2024): 477–495. 10.1016/j.hoc.2023.12.009 38184470

